# Predictive value of the monocyte count for determining the risk of postoperative moderate-to-severe ARDS in patients undergoing one-lung ventilation during radical treatment of esophageal cancer

**DOI:** 10.3389/fmed.2025.1510788

**Published:** 2025-02-11

**Authors:** Feng Zhang, Shunmei Lu, Guilong Wang, Hongyang Xu, Dongxiao Huang, Xiaomin Li

**Affiliations:** ^1^Department of Emergency Medicine, Lianyungang Clinical College of Nanjing Medical University, Lianyungang, China; ^2^Department of Intensive Care Medicine, The Affiliated Wuxi People's Hospital of Nanjing Medical University, Wuxi People's Hospital, Wuxi Medical Center, Nanjing Medical University, Wuxi, China; ^3^Department of Anesthesiology, The Affiliated Wuxi People's Hospital of Nanjing Medical University, Wuxi People's Hospital, Wuxi Medical Center, Nanjing Medical University, Wuxi, Jiangsu, China; ^4^Department of Anesthesiology, Jiangnan University Medical Center, Wuxi, Jiangsu, China

**Keywords:** ARDS, monocyte, esophageal cancer, OLV, predictive, postoperative

## Abstract

**Background:**

This study aimed to screen for risk factors and to assess the predictive value of the monocyte count for the development of moderate-to-severe acute respiratory distress syndrome (ARDS) in patients undergoing one-lung ventilation (OLV) during radical surgery for esophageal cancer.

**Methods:**

In this retrospective study, patients with esophageal cancer admitted to the Department of Thoracic Surgery of Wuxi People’s Hospital between January 2017 and January 2021 were selected. Demographic, preoperative, intraoperative, and postoperative (within 2 h) data were collected. Patients were categorized into moderate-to-severe ARDS and non-moderate-to-severe ARDS groups. Multifactorial logistic regression, receiver operating characteristic (ROC), curve-fitting, and Spearman correlation analysis were used to analyze the data.

**Results:**

After screening, 255 patients were enrolled, with 18% in moderate-to-severe ARDS group. Regression analysis revealed that postoperative monocyte count was an independent predictor for severe ARDS after surgery (OR = 2.916, 95% CI: 1.082–7.863, *p* < 0.05). The optimal cut-off value of postoperative monocyte count in predicting moderate-to-severe ARDS was 0.56 × 10^9^/L (AUC = 0.708) with a sensitivity of 67.4% and a specificity of 66.5%. The difference of predictive value between postoperative monocyte count and prediction model (AUC = 0.760) was not statistically significant (*p* = 0.142). Additionally, a nonlinear connection between postoperative monocyte count and severe ARDS was found using curve fitting.

**Conclusion:**

The postoperative monocyte count is an ideal predictor of postoperative moderate-to-severe ARDS in this patient population and can be used for the early diagnosis of patients with severe postoperative ARDS.

## 1 Introduction

Acute respiratory distress syndrome (ARDS) is characterized by acute diffuse inflammation of lung tissues, edema, and persistent hypoxemia (1). This condition progresses rapidly, is difficult to treat, and has a mortality rate near 50% (2, 3).

Recent global cancer epidemiological statistics show that esophageal tumor representing a serious threat to human health (4). Surgery remains the primary treatment for patients with esophageal tumor; however, lung complications following tumor resection occur in 20–40% of patients, and the incidence of ARDS only follows that of pneumonia and respiratory failure (5). Once patients develop life-threatening adverse respiratory events such as ARDS, the clinical prognosis becomes extremely poor (6). Accordingly, early clinical diagnosis and timely intervention for patients who develop ARDS after esophageal tumor surgery are crucial for improving patient prognosis.

Inflammatory cells play a major role in the development, progression, and metastasis of various cancers (7). For example, Lymphocyte-to-monocyte ratio (LMR), as an inflammatory marker, has been shown to have screening and predicting the prognosis value with different types of malignant tumors. Saeheng’s research suggested that LMR can be treated as a screening factor for intrahepatic cholangiocarcinoma (8). LMR was an valuable predictor of overall survival in patients with resectable pancreatic cancer (9), and it also has good predictive value for pathological complete response in assessing the efficacy of neoadjuvant therapy in breast cancer (10). Patients with esophageal tumor with a low LMR have significantly shorter overall survival (11). Besides, the baseline neutrophil to lymphocyte ratio is the best predictor of post-radiotherapy or chemoradiotherapy outcomes in patients with squamous esophageal cancer (12).

One-lung ventilation (OLV) is a common method of mechanical ventilation during anesthesia for thoracic operations such as radical surgery for esophageal tumor (13), and current research evidence shows that two-lung ventilation does not reduce the incidence of early postoperative pulmonary complications compared with OLV (14). Even lung-protective ventilation strategies are implemented during such operations (15), some patients still develop postoperative ARDS. Mechanical injury to the ventilated lung and hypoxic pulmonary vasoconstriction in the contralateral atrophic lung (16) can lead to the release of inflammatory factors, resulting in damage to the pulmonary capillary endothelium and increased permeability of vascular endothelial cells (17, 18), which in turn can lead to damage to the alveolar-capillary barrier of the lung, contributing to the development of ARDS (19).

Therefore, this study aimed to screen for risk factors including three important subpopulations of white blood cells for the development of moderate-to-severe ARDS in these patients undergoing OLV during radical surgery for esophageal tumor, and to assess the predictive value of monocytes for the risk of developing postoperative moderate-to-severe ARDS according to the new global definition of ARDS.

## 2 Materials and methods

### 2.1 Study participants

In this retrospective study, patients with esophageal tumor admitted to the Department of Thoracic Surgery of Wuxi People’s Hospital between January 2017 and January 2021 were selected. The study was conducted in accordance with medical ethics standards and was approved by the Medical Ethics Committee of Wuxi People’s Hospital, affiliated with Nanjing Medical University (IRB NO: KY-22100). The data are anonymous, and the requirement for informed consent was therefore waived.

### 2.2 Inclusion criteria

The inclusion criteria were as follows: (1) Diagnosis of esophageal tumor confirmed by pathologic tissue; (2) Admitted to the hospital and underwent radical surgery for esophageal tumor under general anesthesia; (3) Underwent intraoperative one-lung ventilation; and (4) Older than 18 years of age.

### 2.3 Exclusion criteria

The exclusion criteria were as follows: (1) Underlying lung disease prior to the operation, including pneumonia, bronchiectasis, asthma, and chronic obstructive pulmonary disease; (2) Comorbid chronic organ insufficiency prior to the operation, including heart failure, chronic kidney disease, chronic liver failure, rheumatic-autoimmune disease, and hematologic disease; (3) Thoracic surgery within 1 year prior to surgery for esophageal malignancies; (4) Sudden intraoperative events that may affect postoperative outcomes; (5) Concomitant operation at other sites during surgery; (6) Presence of anastomotic fistula within 7 days postoperatively; and (7) Major follow-up data missing.

### 2.4 Study groups

The postoperative conditions of patients were comprehensively evaluated based on the diagnostic criteria for the new definition of ARDS (20). The patients whose oxygenation status met the conditions of moderate or severe ARDS for intubation were included in the moderate-to-severe ARDS group; the patients with mild ARDS and patients without ARDS who did not have any hypoxemia and could be extubed immediately after surgery were belonged to the non-moderate to severe ARDS group.

### 2.5 Data collection

The electronic health record system of our hospital was used to collect patient clinical data, including: (1) Demographic data: age, body mass index (BMI), sex, history of coronary heart disease, history of diabetes, and history of hypertension; (2) Preoperative data: white blood cell count, neutrophil count, monocyte count, lymphocyte count, albumin, prealbumin; (3) Intraoperative data: tumor site (upper/middle/lower esophagus), surgical approach (right chest and abdomen/neck, right chest, and abdomen), use of video-assisted thoracoscopy, presence of adhesions between the lungs and pleura, clinical stage of the tumor, intraoperative use of glucocorticoids, intraoperative use of vasopressors (norepinephrine), intraoperative use of antihypertensives (nitroglycerin/urapidil), duration of surgery, duration of OLV, total intraoperative input, intraoperative fluid infusion volume, intraoperative crystalloid fluid infusion volume, intraoperative colloid infusion volume, intraoperative blood transfusion volume, intraoperative red blood cell transfusion volume, intraoperative plasma transfusion volume, total intraoperative output, intraoperative urine output, and intraoperative blood loss; and (4) Postoperative data (blood specimens collected within 2 h postoperatively): white blood cell count, neutrophil count, monocyte count, lymphocyte count, albumin, lactate, C-reactive protein (CRP).

### 2.6 Statistics

SPSS 26.0 (IBM) and R v.4.2.0 software were used for statistical analysis. Quantitative data were tested for normality using the Kolmogorov–Smirnov test. Quantitative data conforming to the normal distribution are expressed as $\bar{X\;}$ ±s, and the independent samples *t*-test was used for comparisons between the two groups. Quantitative data not conforming to the normal distribution are expressed as median and interquartile range [M (QL, QU)], and the Wilcoxon rank-sum test was used for comparisons between the two groups. Count data are expressed as frequencies and percentages [cases (%)], and the Chi-square test (χ^2^) or Fisher’s exact test was used for comparisons between the two groups. Grade data are expressed as frequencies and percentages [cases (%)], and the Wilcoxon rank-sum test was used for the comparison. The white blood cell counts and the counts of its subpopulations and albumin levels were compared between the groups and also within the same patients before and after surgery using either the paired *t*-test or the paired-sample rank-sum test. All parameters were analyzed by univariate binary logistic regression, and parameters with *p* < 0.1 were analyzed further by multivariate binary logistic regression. Based on multivariate logistic regression analysis, a regression equation was constructed by combining the predictors, a receiver operator characteristic (ROC) curve was plotted, and the area under the curve (AUC) was calculated. ROC analysis was used to determine the statistical significance of the differences between the AUC values of the predictive model and postoperative monocyte count. Curve fitting of the association between postoperative monocyte count and risk of moderate-to-severe ARDS was performed using the R package mgcv (v. 1.9.0). Spearman correlation analysis was used to investigate the relationship between duration of OLV and postoperative monocyte count. All tests were two-sided, and differences with *p* < 0.05 were considered statistically significant.

## 3 Results

### 3.1 Screening of study participants

**Figure 1:**
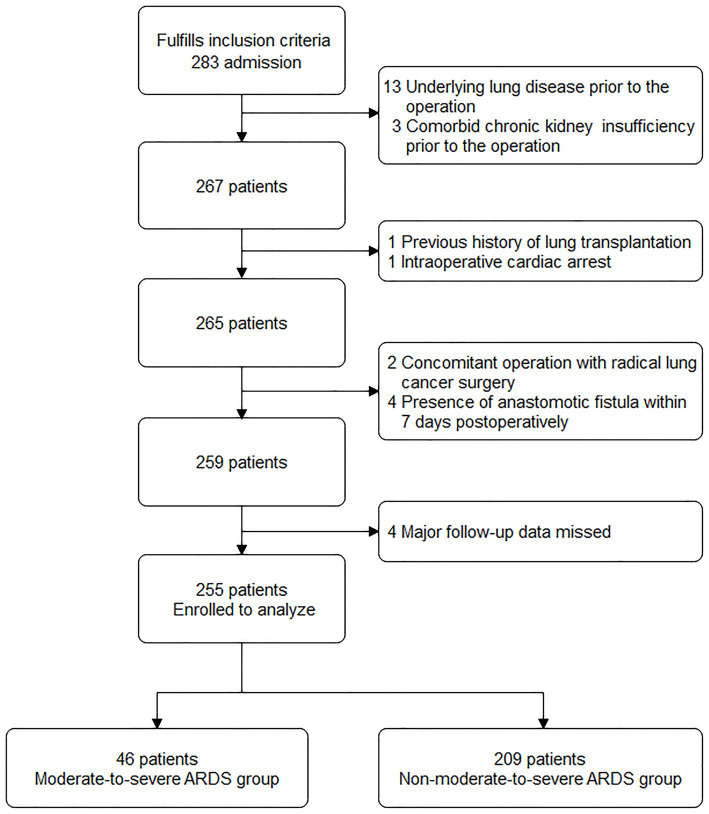
Screening of study participants.

Based on the inclusion criteria, 283 patients were entered into the screening process. After following the screening procedure and applying the exclusion criteria, 255 patients were ultimately analyzed, with 46 patients in the moderate-to-severe ARDS group and 209 in the non-moderate-to-severe ARDS group (Figure 1).

### 3.2 Comparison of clinical characteristics and parameters

A total of 255 patients who underwent radical surgery for esophageal tumor were included in this study, including 245 cases of squamous cell carcinoma, 3 cases of small cell carcinoma, 2 cases of adenocarcinoma, 2 cases of mixed carcinoma, 1 case of sarcomatoid carcinoma, 1 case of lymphoepithelioma-like carcinoma, and 1 case of neuroendocrine carcinoma. The mean age of the population was 67 years, and there were 202 male (79.2%) and 53 female patients (20.8%). The results showed that the difference of thirteen parameters, including postoperative monocyte count between the two groups was statistically significant (*p* < 0.05) (Table 1).

**Table 1:** Comparison of parameters between patients in the moderate-to-severe and non-moderate-to-severe ARDS groups after radical surgery for esophageal cancer.

Variables	Total (*n* = 255)	Non-moderate-to-severe ARDS group (*n* = 209)	Moderate-to-severe ARDS group (*n* = 46)	*p* value
Demographic data				
Age (years)	67 (63, 72)	67 (63, 72)	67.46 ± 6.19	0.639
BMI (kg/m^2^)	22.48 ± 3.09	22.31 ± 3.15	23.23 ± 2.70	0.068
Gender				0.531
Male	202 (79.2%)	164 (78.5%)	38 (82.6%)	
Female	53 (20.8%)	45 (21.5%)	8 (17.4%)	
Coronary heart disease				0.958
No	247 (96.9%)	203 (97.1%)	44 (95.7%)	
Yes	8 (3.1%)	6 (2.9%)	2 (4.3%)	
Diabetes mellitus				0.924
No	231 (90.6%)	190 (90.9%)	41 (89.1%)	
Yes	24 (9.4%)	19 (9.1%)	5 (10.9%)	
Hypertension				0.003
No	154 (60.4%)	135 (64.6%)	19 (41.3%)	
Yes	101 (39.6%)	74 (35.4%)	27 (58.7%)	
Preoperative data				
White blood cell (×10^9/L)	5.86 (4.8, 7.02)	5.7 (4.76, 6.93)	6.67 ± 2.09	0.038
Neutrophil(×10^9/L)	3.53 (2.68, 4.62)	3.48 (2.60, 4.48)	3.75 (2.96, 4.95)	0.105
Monocyte (×10^9/L)	0.46 (0.37, 0.60)	0.46 (0.36, 0.60)	0.47 (0.43, 0.59)	0.099
Lymphocyte (×10^9/L)	1.53 (1.21, 1.90)	1.56 ± 0.51	1.66 ± 0.52	0.226
Albumin (g/L)	38.12 ± 3.64	38.07 ± 3.54	38.33 ± 4.10	0.671
Prealbumin (mg/L)	225.20 (176.70, 271.70)	228.10 ± 70.02	234.99 ± 69.72	0.546
Intraoperative data				
Tumor site				0.996
Upper esophagus	23 (9.0%)	19 (9.1%)	4 (8.7%)	
Middle esophagus	122 (47.8%)	100 (47.8%)	22 (47.8%)	
Lower esophagus	110 (43.1%)	90 (43.1%)	20 (43.5%)	
Surgical approach				0.769
Right chest and abdomen	84 (32.9%)	68 (32.5%)	16 (34.8%)	
Neck, right chest and abdomen	171 (67.1%)	141 (67.5%)	30 (65.2%)	
Video-assisted thoracoscopy				0.387
No	169 (66.3%)	136 (65.1%)	33 (71.7%)	
Yes	86 (33.7%)	73 (34.9%)	13 (28.3%)	
Adhesions between lungs and pleura				0.012
No	210 (82.4%)	178 (85.2%)	32 (69.6%)	
Yes	45 (17.6%)	31 (14.8%)	14 (30.4%)	
Clinical stage of the tumor				0.202
0	2 (0.8%)	2 (1%)	0 (0%)	
I	55 (21.6%)	47 (22.5%)	8 (17.4%)	
II	96 (37.6%)	78 (37.3%)	18 (39.1%)	
III	82 (32.2%)	70 (33.5%)	12 (26.1%)	
IV	20 (7.8%)	12 (5.7%)	8 (17.4%)	
Glucocorticoids				0.089
No	31 (12.2%)	22 (10.5%)	9 (19.6%)	
Yes	224 (87.8%)	187 (89.5%)	37 (80.4%)	
Vasopressors (norepinephrine)				0.779
No	198 (77.6%)	163 (78%)	35 (76.1%)	
Yes	57 (22.4%)	46 (22%)	11 (23.9%)	
Antihypertensives (nitroglycerin/urapidil)				0.641
No	222 (87.1%)	181 (86.6%)	41 (89.1%)	
Yes	33 (12.9%)	28 (13.4%)	5 (10.9%)	
Duration of surgery (min)	230 (195, 295)	230 (191.50, 290)	239 (195.75, 300.25)	0.275
Duration of OLV (min)	103 (81, 135)	96 (77.5, 127)	136.48 ± 54.41	<0.001
Total intraoperative input (ml)	2000 (1750, 2,500)	2000 (1,500, 2,500)	2,425 (2000, 2,525)	0.003
Fluid (ml)	2000 (1750, 2,500)	2000 (1,500, 2,500)	2,175 (2000, 2,500)	0.008
Crystalloid fluid (ml)	1,350 (1,000, 1,500)	1,000 (1,100, 1,500)	1,500 (1,000, 1,500)	0.014
Colloid (ml)	1,000 (500, 1,000)	1,000 (500, 1,000)	1,000 (500, 1,000)	0.102
Blood (ml)	0 (0.0)	0 (0.0)	0 (0.0)	0.015
Red blood cell (ml)	0 (0.0)	0 (0.0)	0 (0.0)	0.014
Plasma (ml)	0 (0.0)	0 (0.0)	0 (0.0)	0.639
Total intraoperative output (ml)	700 (500, 900)	600 (500, 800)	850 (537.5, 1,125)	0.002
Urine (ml)	400 (300, 600)	400 (300, 600)	525 (300, 725)	0.011
Blood loss (ml)	200 (200, 300)	200 (200, 300)	300 (200, 425)	0.004
Postoperative data				
White blood cell (×10^9/L)	12.68 (10.21, 15.60)	12.47 (10.19, 15.18)	13.71 (10.49, 16.62)	0.072
Neutrophil (×10^9/L)	11.33 (8.75, 13.75)	11.14 (8.79, 13.31)	12.48 ± 5.01	0.148
Monocyte (×10^9/L)	0.51 (0.34, 0.68)	0.47 (0.32, 0.63)	0.64 (0.50, 1.00)	<0.001
Lymphocyte (×10^9/L)	0.72 (0.50, 1.13)	0.71 (0.48, 1.10)	0.96 ± 0.53	0.127
Albumin (g/L)	31.91 ± 5.00	32.06 ± 4.79	31.25 ± 5.89	0.322
Lactate (mmol/L)	2.30 (1.60, 3.40)	2.20 (1.60, 3.35)	2.40 (1.50, 3.43)	0.982
CRP (mg/L)	3.20 (1.10, 7.30)	3.20 (1.00, 6.70)	3.95 (1.78, 10.73)	0.086

ARDS, acute respiratory distress syndrome; BMI, body mass index; CRP, C-reactive protein.

### 3.3 Comparison of the white blood cell count and counts of its subpopulations and albumin before and after the operation

The white blood cell counts and the counts of its subpopulations, albumin, and other parameters of all patients were compared within subjects before and after surgery. Only the non-moderate-to-severe ARDS group had no statistically significant difference between the preoperative and postoperative monocyte count (*p* > 0.05). Therefore, the operation was not believed to affect monocyte count in the non-ARDS group. In contrast, the differences in the remaining parameters of this group as well as in all parameters in the other two groups before and after the operation were statistically significant (*p* < 0.05) (Table 2).

**Table 2:** Comparison of the white blood cell count and counts of its subpopulations and albumin before and after the operation in all patients and between the two groups.

Variables	Before the operation	After the operation	*t/Z* value	*p* value
Total (*n* = 255)				
White blood cell (×10^9/L)	5.86 (4.80, 7.02)	12.68 (10.21, 15.60)	−13.79	<0.001
Neutrophil(×10^9/L)	3.53 (2.68, 4.62)	11.33 (8.75, 13.75)	−13.84	<0.001
Monocyte (×10^9/L)	0.46 (0.37, 0.60)	0.51 (0.34, 0.68)	−2.16	0.031
Lymphocyte (×10^9/L)	1.53 (1.21, 1.90)	0.72 (0.50, 1.13)	−11.50	<0.001
Albumin (g/L)	38.12 ± 3.64	31.91 ± 5.00	17.52	<0.001
Moderate-to-severe ARDS group (*n* = 46)				
White blood cell (×10^9/L)	6.67 ± 2.09	13.71 (10.49, 16.62)	−5.88	<0.001
Neutrophil(×10^9/L)	3.75 (2.96, 4.95)	12.48 ± 5.01	−10.99	<0.001
Monocyte (×10^9/L)	0.47 (0.43, 0.59)	0.64 (0.50, 1.00)	−4.15	<0.001
Lymphocyte (×10^9/L)	1.66 ± 0.52	0.96 ± 0.53	8.725	<0.001
Albumin (g/L)	38.33 ± 4.10	31.25 ± 5.89	−5.45	<0.001
Non-moderate-to-severe ARDS group (*n* = 209)				
White blood cell (×10^9/L)	5.70 (4.76, 6.93)	12.47 (10.19, 15.18)	−12.49	<0.001
Neutrophil(×10^9/L)	3.48 (2.60, 4.48)	11.14 (8.79, 13.31)	−12.54	<0.001
Monocyte (×10^9/L)	0.46 (0.36, 0.60)	0.47 (0.32, 0.63)	−0.57	0.568
Lymphocyte (×10^9/L)	1.56 ± 0.51	0.71 (0.48, 1.10)	−10.20	<0.001
Albumin (g/L)	38.07 ± 3.54	32.06 ± 4.79	16.67	<0.001

### 3.4 Univariate logistic regression analysis of factors influencing moderate-to-severe ARDS

Eighteen parameters showed statistically significant in the univariate regression analysis, including postoperative monocyte count (*p* < 0.1), with it having the highest OR of 4.288 (Table 3). Although these results have not yet been controlled for multiple confounders, they suggest that postoperative monocyte count plays some role in predicting the risk of postoperative moderate-to-severe ARDS.

**Table 3:** Univariate logistic regression analysis of factors influencing moderate-to-severe ARDS after radical surgery for esophageal cancer.

Variables	OR value	95%CI	*p* value
Demographic data			
Age (years)	1.016	0.969–1.065	0.517
BMI (kg/m2)	1.099	0.992–1.217	0.070
Gender	0.767	0.334–0.761	0.532
Coronary heart disease	1.538	0.300–7.874	0.605
Diabetes mellitus	1.220	0.430–3.455	0.709
Hypertension	2.592	1.351–4.975	0.004
Preoperative data			
White blood cell (×10^9/L)	1.165	1.001–1.355	0.048
Neutrophil(×10^9/L)	1.156	0.976–1.368	0.093
Monocyte (×10^9/L)	2.382	0.473–11.994	0.292
Lymphocyte (×10^9/L)	1.458	0.792–2.685	0.226
Albumin (g/L)	1.019	0.993–1.113	0.670
Prealbumin (mg/L)	1.001	0.997–1.006	0.545
Intraoperative data			
Tumor site			
Upper esophagus (Reference)	1.000		
Middle esophagus	0.947	0.291–3.089	0.929
Lower esophagus	0.990	0.507–1.933	0.977
Surgical approach	0.904	0.462–1.771	0.769
Video-assisted thoracoscopy	0.734	0.364–1.481	0.388
Adhesions between lungs and pleura	2.512	1.205–5.238	0.014
Clinical stage of the tumor	1.324	0.927–1.889	0.123
Glucocorticoids	0.484	0.206–1.134	0.095
Vasopressors (norepinephrine)	1.114	0.525–2.363	0.779
Antihypertensives (nitroglycerin/urapidil)	0.788	0.287–2.165	0.644
Duration of surgery (min)	1.003	0.098–1.008	0.205
Duration of OLV (min)	1.012	1.005–1.018	<0.001
Total intraoperative input (ml)	1.001	1.000–1.001	0.002
Fluid (ml)	1.001	1.000–1.001	0.008
Crystalloid fluid (ml)	1.001	1.000–1.002	0.051
Colloid (ml)	1.001	1.000–1.002	0.053
Blood (ml)	1.002	1.000–1.004	0.049
Red blood cell (ml)	1.002	1.000–1.004	0.018
Plasma (ml)	0.963	0.000–1.000	1.000
Total intraoperative output (ml)	1.001	1.000–1.002	0.001
Urine (ml)	1.001	1.000–1.002	0.014
Blood loss (ml)	1.002	1.001–1.004	0.007
Postoperative data			
White blood cell (×10^9/L)	1.056	0.991–1.126	0.093
Neutrophil (×10^9/L)	1.053	0.983–1.128	0.139
Monocyte (×10^9/L)	4.288	1.602–11.480	0.004
Lymphocyte (×10^9/L)	1.183	0.714–1.960	0.514
Albumin (g/L)	0.968	0.909–1.032	0.321
Lactate (mmol/L)	1.089	0.941–1.261	0.251
CRP (mg/L)	0.998	0.984–1.012	0.821

ARDS, acute respiratory distress syndrome; BMI, body mass index; CRP, C-reactive protein.

### 3.5 Multifactorial logistic regression analysis of risk factors for moderate-to-severe ARDS

**Figure 2:**
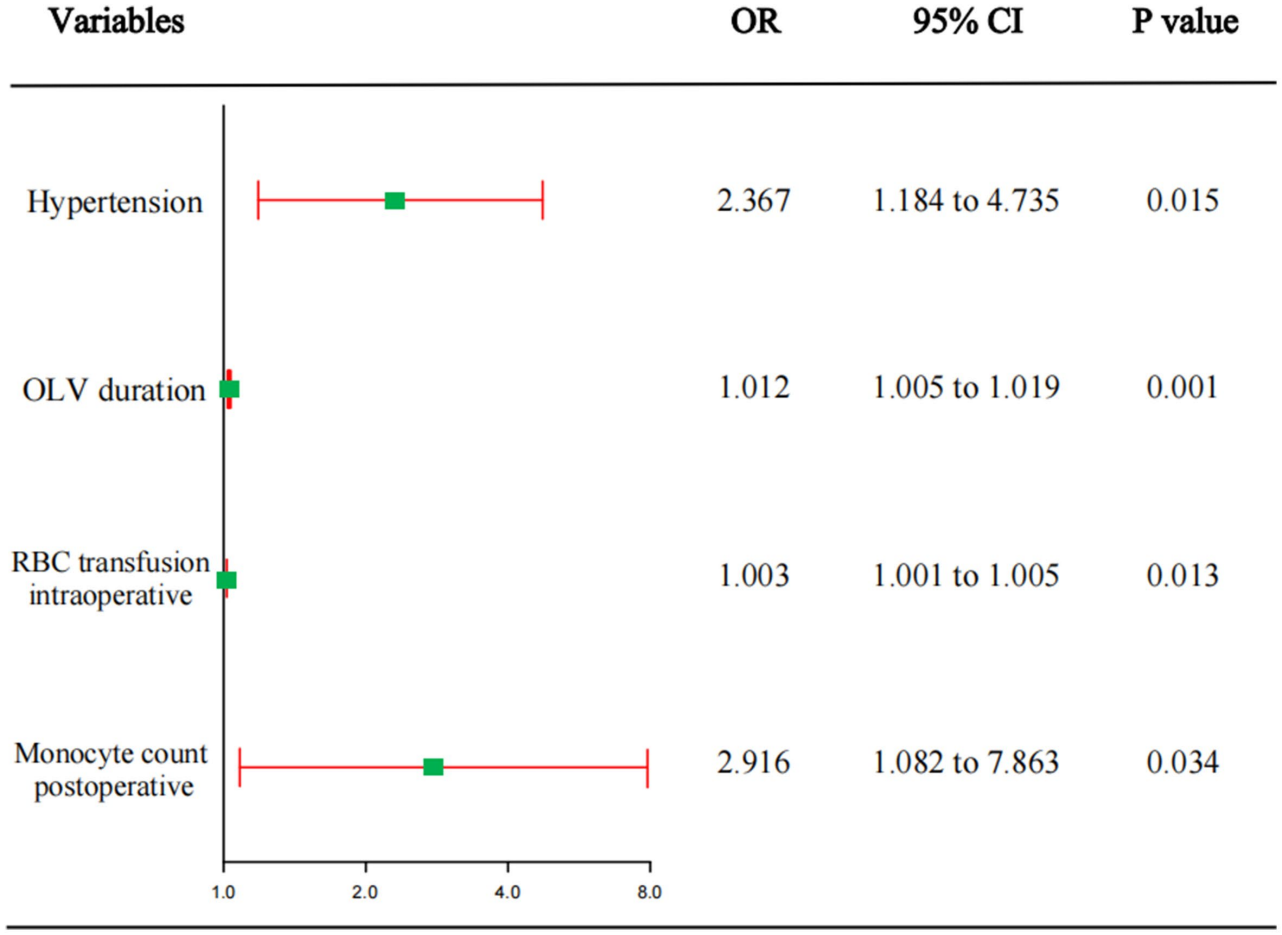
Multifactorial logistic regression analysis of risk factors for moderate-to-severe ARDS.

Univariate logistic regression analysis of each influencing factor was performed. To avoid missing important predictors, *p* < 0.1 was used as the criterion for judgment. In total, eighteen parameters above, including postoperative monocyte count were incorporated into the multivariate logistic regression analysis. After controlling for multiple confounders, regression analysis showed that hypertension, duration of OLV, intraoperative red blood cell transfusion volume, and postoperative monocyte count were independent risk factors for the development of moderate-to-severe ARDS after radical surgery for esophageal tumor (Figure 2).

### 3.6 Construction of a joint prediction model and assessment of its predictive power for risk factors

Based on the multifactorial logistic regression analysis, four indicators (hypertension, duration of OLV, intraoperative red blood cell transfusion, and postoperative monocyte count) were combined to construct a joint prediction model: logit (P) = −4.051 + 0.862 × hypertension+0.011 × duration of OLV + 0.003 × intraoperative red blood cell transfusion+1.070 × postoperative monocyte count.

**Figure 3:**
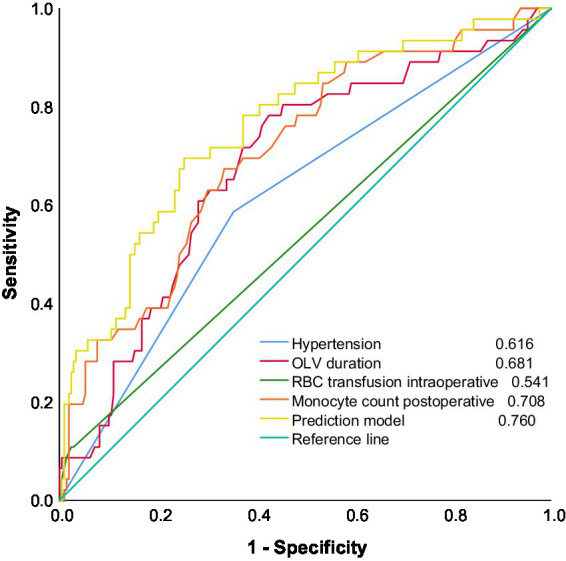
Early risk prediction model for patients with moderate-to-severe ARDS after radical surgery for esophageal cancer and ROC curves for each predictor.

In addition, the joint prediction model was used as a new variable to construct ROC curves together with the four predictors entered into the model to assess the magnitude of their respective predictive powers (Figure 3). The “Youden index” was used to determine the cutoff values, sensitivity, and specificity of four indicators, and the joint prediction model in the prediction of moderate-to-severe ARDS (Table 4).

**Table 4:** Risk prediction model for patients with moderate-to-severe ARDS after radical surgery for esophageal cancer with the predictive value for each risk factor.

Variables	AUC	95%CI	cutoff value	Sensitivity (%)	Specificity (%)
Hypertension	0.616	0.526–0.707	–	–	–
Duration of OLV (min)	0.681	0.596–0.766	104.5	78.3	57.4
intraoperative Red blood cell input (ml)	0.541	0.444–0.637	350	10.9	97.6
Postoperative Monocyte (×10^9/L)	0.708	0.626–0.790	0.56	67.4	66.5
Prediction mode (%)	0.760	0.682–0.838	18.3	69.6	74.6

ARDS, acute respiratory distress syndrome; AUC, area under the receiver operating characteristic curve; 95% CI, 95% confidence interval.

### 3.7 Comparison of the difference between the AUC values of the predictive model and postoperative monocyte count

Given that the AUCs of the postoperative monocyte count and the predictive model were both greater than 0.7 and extremely close (Figure 3), ROC analysis was used to determine the statistical significance of the difference between the AUC of the predictive model and the postoperative monocyte count. The results showed that *Z* = 1.469 and *p* = 0.142, indicating that the difference between the two models was not statistically significant (*p* > 0.05).

### 3.8 Analysis of the association between the postoperative monocyte count and the risk of postoperative moderate-to-severe ARDS

**Figure 4:**
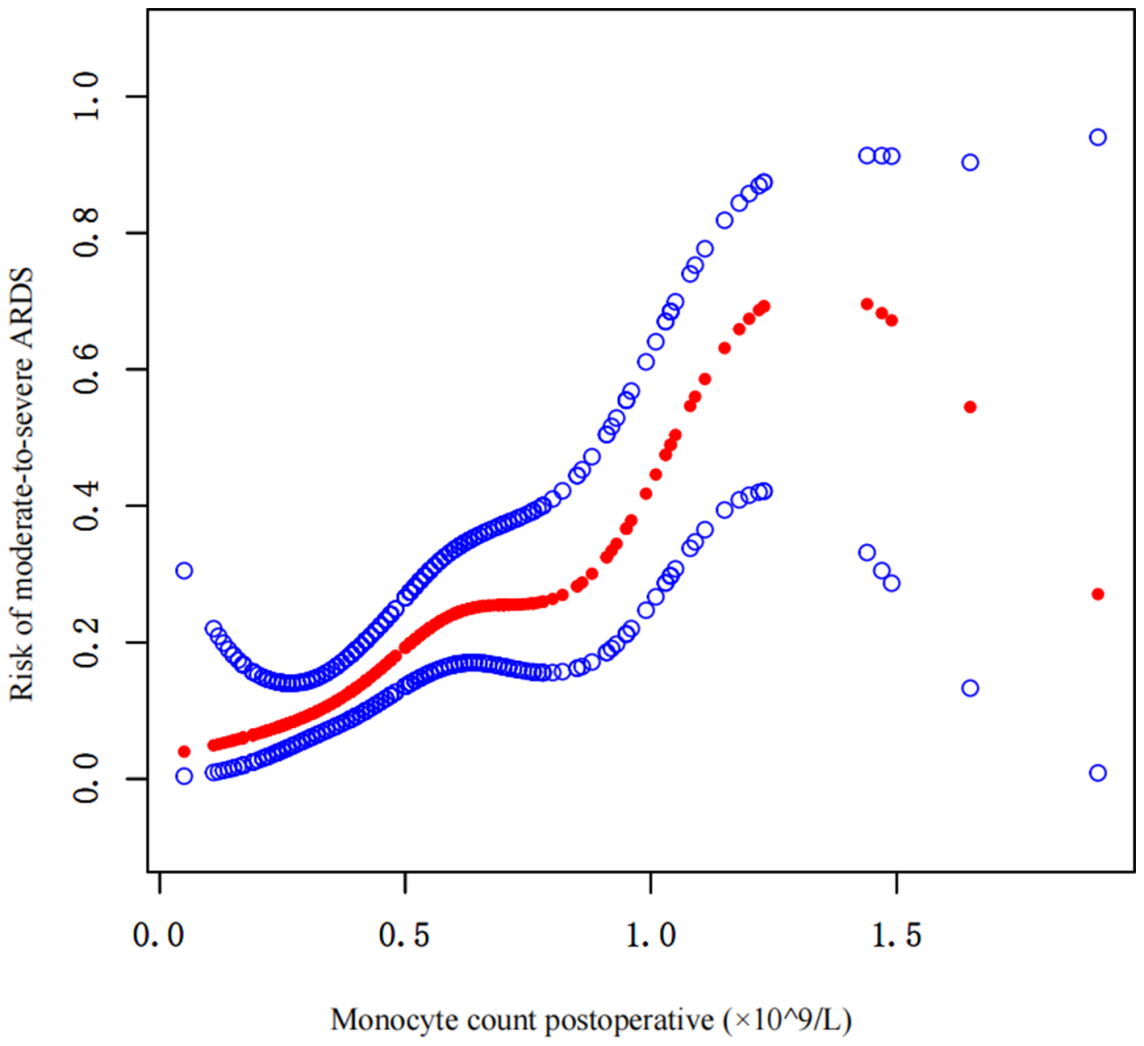
Curve fitting of the correlation between the postoperative monocyte count and risk of postoperative moderate-to-severe ARDS.

Curve fitting was used to investigate the correlation between the postoperative monocyte count and the risk of moderate-to-severe ARDS. The results revealed a significant correlation between the two, especially when the postoperative monocyte count was between the upper limit of the reference range of 0.8 × 10^9^/L and 1.25 × 10^9^/L. As the monocyte count increased, the risk of postoperative moderate-to-severe ARDS increased sharply (Figure 4).

### 3.9 Stratified analysis of the postoperative monocyte count for predicting the risk of postoperative moderate-to-severe ARDS

To assess the stability of the postoperative monocyte count for predicting the risk of postoperative moderate-to-severe ARDS as well as to discover special patient populations, the patients were stratified by three common adjustment variables (sex, tumor site, and type of surgical procedure). The postoperative monocyte count was statistically significant (*p* < 0.05) for predicting the risk of postoperative moderate-to-severe ARDS in four population subgroups, with male, female, tumor site (lower esophagus), and surgical approach (neck-right chest-abdomen) (Table 5).

**Table 5:** Subgroup analysis of the effect of the postoperative monocyte count on postoperative moderate-to-severe ARDS after radical surgery for esophageal cancer.

Subgroup	Number	OR value	95%CI	*p* value
Gender				
Male	202	3.49	(1.29, 9.43)	0.014
Female	53	46.38	(1.04, 2073.90)	0.048
Tumor site				
Upper esophagus	23	1.51	(0.00, 500.15)	0.889
Middle esophagus	122	2.49	(0.78, 7.87)	0.122
Lower esophagus	110	7.86	(1.98, 31.25)	0.003
Surgical approach				
Right chest and abdomen	84	3.05	(0.80, 11.64)	0.102
Neck, right chest and abdomen	171	5.54	(1.54, 19.93)	0.009

ARDS, acute respiratory distress syndrome.

### 3.10 Analysis of the correlation between the duration of OLV and postoperative monocyte count

**Figure 5:**
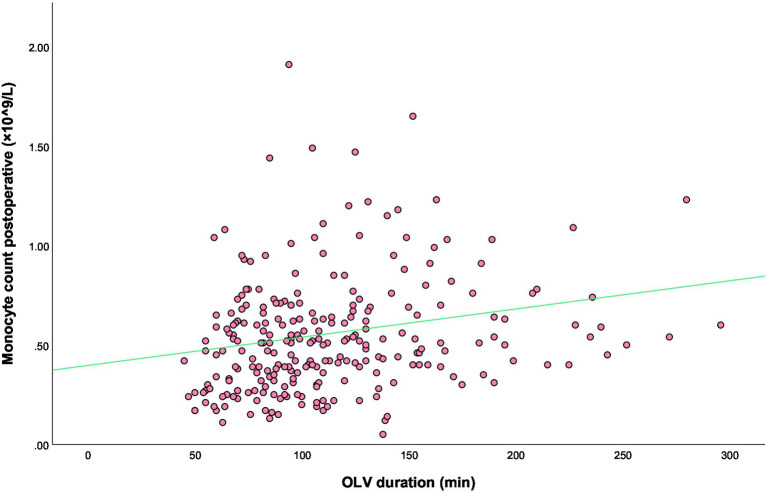
Scatterplot of the best-fit line for the correlation between the duration of OLV and postoperative monocyte count.

OLV can lead to mechanical ventilation-associated lung injury. The results of the present study suggest that the duration of OLV and postoperative monocyte count are both independent risk factors for postoperative moderate-to-severe ARDS. To further investigate the effect of OLV on the postoperative monocyte count and analyze the relationship between the two, we first analyzed the scatter plots of this relationship, and then performed Spearman correlation analysis. The results showed a positive linear correlation between the two (Figure 5), with a correlation coefficient of *r* = 0.262 (*p* < 0.001) and a coefficient of determination (*R*^2^) of 0.032.

## 4 Discussion

Esophageal tumor surgery under OLV had a significant effect on major inflammatory cell types as well as albumin, in particular by increasing the neutrophil count and decreasing the lymphocyte count. The postoperative monocyte count in the moderate-to-severe ARDS group was significantly higher than preoperative counts, while there was no significant change in the control group. There was also a statistically significant difference in the postoperative monocyte count between the two groups, with the moderate-to-severe ARDS group having higher counts, suggesting that the elevated monocyte counts were strongly associated with postoperative moderate-to-severe ARDS.

Inflammatory monocytes play a major role in the pro-inflammatory process of ventilator-induced lung injury (VILI) (21, 22). During lung ischemia–reperfusion injury, monocytes mediate neutrophil infiltration into lung tissue and exacerbate lung injury (23, 24). Monitoring the inflammatory response following the infection of juvenile mice with influenza A virus showed that inflammatory monocytes were over-recruited in the lungs and mediated an excessive inflammatory response, thereby damaging lung tissues. In addition, the degree of inflammation stimulated in the lungs was not correlated with the initial viral titer administered, suggesting that inflammatory monocytes are critical for secondary lung injury (25). Animal experiments and high-throughput mass cytometry by Xu et al. demonstrated that inducing apoptosis of lung monocyte-derived macrophages using an immunosuppressive transmembrane protein could reduce inflammatory injury in the lungs and improve the survival rate in a mouse model of ARDS, and that the protective effect of the protein on ARDS mice was significantly weakened with monocyte/macrophage depletion, suggesting that monocytes are the target cells for anti-inflammatory treatment of ARDS (26). In summary, monocytes play an important role in the inflammatory response leading to lung injury/ARDS. These speculations were confirmed by our logistic regression and ROC curve analyses. The postoperative monocyte count was an independent factor with high predictive value for predicting postoperative severe ARDS. Its predictive value was slightly lower than that of the joint prediction model; however, ROC analysis results showed that there was no significant difference between the two with respect to predictive value. It means that the AUC value of the predictive model exhibited no significant advantage. Overall, the predictive model is relatively less practical. Thus, the postoperative monocyte count is an ideal predictor for predicting the development of moderate-to-severe ARDS following radical surgery for esophageal tumor under OLV. Curve fitting was used to investigate the correlation between postoperative monocyte count and the risk of moderate-to-severe ARDS, and the results suggested a significant correlation between the two, especially at specific intervals; as the monocyte count increased, the risk of developing moderate-to-severe ARDS increased.

In the stratified analysis of the present study, the results of the subgroup analysis of female patients were not included due to the large OR and 95% confidence intervals, which may be due to the instability of the OR caused by the small number of female patients. The ORs for male sex, lower esophageal tumor site, and surgical approach (neck-right chest-abdomen) were higher than the overall OR, suggesting that the risk of severe ARDS associated with an increased postoperative monocyte count was higher in these three subpopulations. These results suggest that monocyte count has a greater impact on ARDS in male patients, which may be due to two reasons. In the literature, the risk of monocyte-mediated disorders in males has been evaluated from the perspective of androgens. Supplemental testosterone regimens have been shown to lead to a pro-inflammatory response in classical monocytes in both humans and mice, and plasma testosterone levels in individuals undergoing sex reassignment surgery are positively correlated with pro-inflammatory cytokine and chemokine secretion from cultured peripheral monocytes following stimulation, confirming the hypothesis that androgens may increase the risk of monocyte-mediated disorders in males (27). In addition to increasing the pro-inflammatory response of monocytes, androgens promote monocyte development during bone marrow hematopoiesis, leading to increased differentiation of monocytes in the bone marrow and higher monocyte counts in the peripheral blood in males than in females (28). The ORs of the patient subpopulations with tumors in the lower esophagus and who were operated via the neck-right chest-abdomen approach were higher than those of the other groups. No relevant literature reports were found to reasonably explain these phenomena, which is presumed to be related to the more intense inflammatory reaction caused by the greater trauma of the surgery. However, the specific reasons need to be further investigated.

Our study focused on the prediction value of postoperative monocyte count in severe ARDS after radical treatment of esophageal cancer, and on the basis of multivariate Logistic regression analysis, The prediction model of ARDS was further constructed by combining hypertension, duration of OLV, intraoperative red blood cell input and postoperative monocyte count. Although ROC analysis showed no statistical difference between the predictive model AUC and the postoperative monocyte AUC, the predictive model performs well statistically. If the advantage of simplicity of monocyte count is ignored, we can collect these four parameters, and substitute them into the prediction regression equation to calculate the risk probability of postoperative ARDS of the patient (similar to our routine nutritional risk screening after surgery). In order to give early warning to clinicians, early implementation of preventive measures (such as control of fluid intake, active anti-inflammatory therapy, etc.) to reduce the harm that may be brought by ARDS in the later stage.

This is a single-center clinical retrospective study. Accordingly, future multi-center clinical studies will further increase the reliability of the findings. In addition, the present study focused on lung injury associated with radical surgery for esophageal tumor under OLV. More clinical studies are needed to verify whether these findings are applicable to other types of lung injury (e.g., sepsis-associated lung injury, transfusion-associated lung injury). Furthermore, LMR has been shown to be useful in predicting the effects of simultaneous radiotherapy and chemotherapy in patients with esophageal tumors (29), and it was also useful in identifying patients with a poor prognosis after radical esophagectomy (30). Whether this combined index is better than monocyte count in predicting the risk of developing severe ARDS needs to be further explored.

## 5 Conclusion

The monocyte count in the early postoperative period following radical surgery for esophageal tumor is an ideal predictor of moderate-to-severe postoperative ARDS and a simple clinical indicator for assessing the intensity of the “inflammatory storm” during OLV-associated lung injury.

## Data Availability

The data analyzed in this study is subject to the following licenses/restrictions: in this retrospective study, patients with esophageal cancer admitted to the Department of Thoracic Surgery of Wuxi People’s Hospital between January 2017 and January 2021 were selected. Demographic, preoperative, intraoperative, and postoperative (within 2 h) data were collected. Requests to access these datasets should be directed to Xiaomin Li, lyglxm1@163.com.
